# Suboptimal outcomes after closed reduction and internal fixation of displaced femoral neck fractures in middle-aged patients: is internal fixation adequate in this age group?

**DOI:** 10.1186/s12891-018-2120-9

**Published:** 2018-06-09

**Authors:** Cheng-Tzu Wang, Jia-Wan Chen, Karl Wu, Chiang-Sang Chen, Wen-Chih Chen, Jwo-Luen Pao, Chih-Hung Chang, Tsung-Yu Lan

**Affiliations:** 1 Department of Orthopaedic Surgery, Far Eastern Memorial Hospital, No. 21, Sec. 2, Nanya S. Rd., Banciao Dist, New Taipei City, 220 Taiwan; 2 Department of Mechanical Engineering, Oriental Institute of Technology, New Taipei City, Taiwan

**Keywords:** Femoral neck fracture, Internal fixation, Middle-aged patients

## Abstract

**Background:**

There have been many studies regarding nongeriatric femoral neck fractures (FNFs), which included patients of a wide age range (between 20 and 60 years old). We aimed to determine whether internal fixation provided acceptable outcomes for middle-aged patients with displaced FNFs, and identify predictors of successful internal fixation.

**Methods:**

A total of 117 patients, aged 50–60 years and who underwent closed reduction and unilateral internal fixation using cannulated screws, were included. The outcomes were classified as either “complications” (varus malunion, femoral neck shortening, non-union/early collapse, avascular necrosis, or arthroplasty during the follow-up) or “optimal outcomes” (no complications). Patients with displaced FNFs (Garden stages III–IV, *n* = 69) were categorized according to whether they experienced acceptable or unacceptable reduction. We evaluated whether patients’ clinical characteristics could predict optimal outcomes.

**Results:**

Patients with displaced FNFs generally experienced complications (84.1%). Twenty-two percent of patients experienced optimal outcomes when acceptable reduction was achieved. Patients with unacceptable reductions experienced complications. Optimal outcomes were positively associated with Pauwels’ type II fracture (OR: 8.67, *p* = 0.025) and negatively associated with excessive alcohol consumption (*p* = 0.045).

**Conclusions:**

Compared with the younger age group, complication rates are higher in middle-aged patients with displaced FNFs treated using cannulated screws. If internal fixation is to be used for a displaced FNF, patient selection is essential. Care must be taken to avoid selecting patients with excessive alcohol consumption, while successful internal fixation may be more likely for patients with Pauwels’ type II fracture.

## Background

Femoral neck fractures (FNFs) are an important health concern, with multiple causes, such as trauma, malignancy, and bone metabolism disorders. The possibility of stress fractures should be especially considered in athletes and recreational runners without a history of trauma, as the rare fracture pattern of FNFs might easily be missed on radiography and is best detected by magnetic resonance imaging (MRI) [1, 2]. Displaced FNFs are difficult to treat, which result in a high rate of complications (e.g., non-union and osteonecrosis) [3, 4]. Age has traditionally been considered an important factor for treatment selection, as closed or open reduction with internal fixation is favored for younger patients, because it preserves the femoral head and cartilage. In contrast, primary arthroplasty is preferred to allow elderly patients to regain their daily functioning [5]. However, the optimal treatment for middle-aged patients is unclear as existing comorbidities and osteoporosis must be considered. Previous studies regarding nongeriatric FNFs included patients with a wide age range, between 20 and 60 years [3, 6–8]. We hypothesized that it might mimic the higher failure rate of middle-aged patients. In addition, national health policies and insurance coverage can significantly influence clinical decision-making. Thus, treatment of FNFs in middle-aged patients is complicated, and only a few studies have evaluated the outcomes of displaced FNFs treated using internal fixation. Therefore, this study aimed to evaluate the outcomes of displaced FNFs after closed reduction and internal fixation among middle-aged patients, and to identify factors that predict successful internal fixation and optimal outcomes.

## Methods

### Patients

This single-center retrospective study evaluated data from 161 middle-aged patients (50–60 years old) with isolated FNFs that were treated using cannulated screws between 2005 and 2014. Since there has been no consensus about the optimal implant for displaced FNFs, patients treated with other implants, including sliding-hip screw (22 patients) and Kowles pins (10 patients) were not included. We hope that simplification of the implant will minimize the selection bias. The patients’ medical records were used to collect data regarding age, sex, mechanism of injury, comorbidities, smoking, and alcohol use.

DSM-IV-TR (Diagnostic and Statistical Manual of Mental Disorders, 4th Edition, Text Revision) criteria were applied for evaluation of alcohol use disorder. In our cohort, patients who met the criteria for “alcohol abuse” or “alcohol dependence” were categorized under “excessive alcohol consumption” [9]. Patients with (1) a delayed surgery > 6 h after diagnosis; (2) malignancy or a pathological fracture; (3) a bone metabolism disorder that could affect fracture healing; (4) end-stage renal disease; (5) multiple fractures requiring ipsilateral femoral shaft fixation; (6) a follow-up period < 2 years; and (7) inappropriate or incomplete radiographs were excluded. Thus, 117 patients were considered eligible for the present study.

### Surgery

Fractures were classified as undisplaced (Garden stages I–II, *n* = 48) or displaced (Garden stages III–IV, *n* = 69). We used Pauwels’ classification to determine whether vertical shearing force significantly influenced internal fixation failure for displaced fractures. All fractures were managed by senior attending surgeons or fellowship-trained orthopedic traumatologists. Patients underwent closed reduction and unilateral internal fixation with cannulated screws, using the lateral approach through a small incision. The first implant was inserted inferiorly along the calcar, and the two other implants were placed superiorly to create a triangular configuration. The implants were inserted deep enough to ensure stability through subchondral bone purchase. The distance between the screw tip and the femoral head border was < 10 mm in all cases (8.1 ± 3.5 mm). Surgeries were performed as soon as possible after the diagnosis, generally within < 6 h at our center. To prevent the bias of delayed surgery, the cases involving surgery > 6 h after the diagnosis were excluded from the present study. Open reduction or capsulotomy was not performed. The patients were not allowed to bear weight for ≥8 weeks after the surgery until they exhibited radiographic evidence of callus formation. After evidence of bony union was detected, touch-down weight bearing with a walker was initiated and the level of ambulation was gradually increased. Follow-up radiography was performed at least twice during the first postoperative month, and then the frequency was decreased to monthly. Patients were followed up for ≥2 years (range: 2–8 years).

### Radiographic Assessment & Outcomes

Preoperative radiographs were taken in patients at the time of admission; the immediate postoperative film was obtained within hours after surgery; and follow-up images were taken in every visit in our outpatient department. Fracture union was defined as a visible callus bridging the fracture site present within 6 months of surgery [6].

Each radiograph was evaluated twice in a blinded fashion by a single author (CT Wang); then, an additional senior staff (TY Lan) repeated the assessments to ensure reliability. To resolve concerns with inter-observer reliability, we used SPSS (Version 22, SPSS Inc., Chicago, Illinois) to determine the intra-class correlation coefficient.

The outcomes were classified as either “optimal outcomes” or “complications”. Optimal outcomes were defined as bony union with < 5° varus and < 5 mm femoral neck shortening [7, 8, 10], osteonecrosis, and subsequent arthroplasty. Complications were defined as > 5° varus malunion or shortening > 5 mm of the femoral neck [7, 8, 10], non-union/early collapse, avascular necrosis (AVN) of the femoral head/neck after union, or arthroplasty during follow-up. Varus malunion and femoral neck shortening were evaluated based on a comparison to the radiographs of the contralateral hip. Regarding the quantitative assessment of femoral neck shortening, we used the method described by Zlowodzki et al. [7, 10]. Because the degree of shortening or varus collapse may change during the follow-up period, assessment was performed on post-operative films, but the last follow-up radiograph (at least 2 years after surgery) was used for the final decision.

Non-union/early collapse was defined as re-displacement of the fracture site requiring an additional surgery during any follow-up radiographic examination, or the absence of radiographic evidence of union at 6 months after surgery [6, 10]. AVN was identified using magnetic resonance imaging or radiography and the advanced Ficat stages (Ficat III/IV).

We counted all complications in patients with co-existing conditions, such as AVN in a patient with varus malunion, non-union/early collapse requiring arthroplasty to regain mobility, or arthroplasty to address osteoarthritis several years after varus malunion.

### Data analysis

The data were categorized according to reduction quality, and the displaced group was divided into a group with acceptable reduction (*n* = 50) or unacceptable reduction (*n* = 19), based on the immediate postoperative radiographs. Acceptable reduction was defined as < 5 mm displacement on the antero-posterior and lateral plain radiograph, ≤5° varus to 25° valgus on the antero-posterior plain radiograph, and ≤ 10° on the lateral plain radiograph [6, 11].

We also evaluated the acceptable reduction group to observe any differences according to Pauwels’ type, smoking, alcohol consumption, or diabetes mellitus, and to identify predictors of optimal outcomes. Variables were compared using the Chi-square test or Fisher’s exact test, as appropriate, and *p*-values of < 0.05 were considered statistically significant. We used SPSS software (Version 22, SPSS Inc., Chicago, Illinois) throughout.

## Results

Patients had a mean age of 55.4 years (range: 50–60 years) and included 43 men (36.7%). Forty-eight patients had Garden stages I–II (the non-displaced group), and 69 patients had Garden stages III–IV (the displaced group) (Table 1). The mechanisms of injury were simple fall in 72 patients, fall-from-height in 28 patients, and vehicle accident in 17 patients. There was no statistical difference in gender and mechanism of injury between the groups.

**Table 1:** The outcomes and complications according to the Garden classification

	Non-displaced	Displaced	*P*-value^*^
All patients (male:female)	48 (13:35)	69 (30:39)	N/A
Optimal outcomes	45 (93.7%)	11 (15.9%)	0.001
Complications	3 (6.3%)	58 (84.1%)	
Varus malunion/shortening	0	27 (39.1%)	0.001
Avascular necrosis	1 (2.1%)	10 (14.5%)	0.026
Non-union/early collapse	2 (4.2%)	23 (33.3%)	0.001
Shift to arthroplasty	3 (6.3%)	26 (37.7%)	0.001

^*^P-values were calculated using Fisher’s exact test

The non-displaced group had an extremely high success rate (93.8%, *n* = 40), with only 2 cases of non-union/early collapse and 1 case that developed osteonecrosis after bony union. No varus malunion or femoral neck shortening was identified in the undisplaced group.

The displaced group had a high complication rate, and optimal outcomes were only achieved in 11 patients (15.9%). The complications during a minimum follow-up of 2 years included non-union/early collapse (33.3%, *n* = 23) and AVN after bony union (14.5%, *n* = 10). Many cases were converted to arthroplasty (37.7%, *n* = 26) because of major complications, which typically involved non-union/early collapse early after surgery or the progression of AVN later after surgery. The displaced group had a union rate of 66.7%, although most of these cases developed femoral neck shortening or varus malunion (27 patients, 39.1% of the displaced group). Two patients underwent revisional ORIF and, subsequently, achieved bony union with varus and shortening.

Acceptable reduction was achieved in 72.5% (*n* = 50) of the cases with displaced fractures (Table 2), although only 22% of these patients (*n* = 11) experienced anatomical union without early collapse or AVN. Thus, the complication rate was 78% when good reduction was initially achieved. Varus malunion or femoral neck shortening occurred in 15 patients, and 6 patients initially had anatomical union but, subsequently, developed AVN after several years (mean: 2 years, range: 1–6 years). Subsequent arthroplasty was performed in 18 patients, which accounted for 36% of patients who achieved acceptable reduction but, eventually, required arthroplasty. Seventeen of these cases involved non-union/early collapse.

**Table 2:** Characteristics of the displaced group according to reduction quality

	Acceptable	Unacceptable	*P*-value^*^
All patients (male:female)	50 (22:28)	19 (8:11)	N/A
Optimal outcomes	11 (22%)	0	0.028
Complications	39 (78%)	19 (100%)	
Varus malunion/shortening	15 (30%)	12 (63.2%)	0.007^†^
Avascular necrosis	8 (16%)	2 (10.5%)	0.715
Non-union/early collapse	17 (34%)	6 (31.5%)	0.773^†^
Shift to arthroplasty	18 (36%)	8 (42.1%)	0.640^†^

^*^P-values calculated using the chi-square test

^†^*P*-values calculated using the Fisher’s exact test

None of the patients in the unacceptable reduction group achieved optimal outcomes, and this outcome was significantly poorer than the optimal rate of 22% in the acceptable group (*p* = 0.028). The varus malunion rate was significantly higher in the unacceptable reduction group (63.2% vs. 30%, *p* = 0.007). The rates of AVN with subsequent arthroplasty were high in both groups, and the difference was not statistically significant (*p* = 0.715) (Table 2).

Most patients who achieved optimal outcomes in the acceptable reduction group had Pauwels’ type II fractures (91%). The odds ratio for optimal outcomes among Pauwels’ type II fractures was 8.67 (*p* = 0.025) (Table 3). Patients with complications were more likely to smoke (25.6%), excessively consume alcohol (28.2%), and have diabetes mellitus (17.9%), compared to patients that did not experience complications. Only excessive alcohol consumption significantly predicted poor outcomes in the present study (*p* = 0.045) (Table 4).

**Table 3:** Outcomes in the acceptable reduction group according to Pauwels’ classification

	Optimal outcomes	Complications
Pauwels’ I	0	4 (100%)
Pauwels’ II	10 (34.5%)^*^	19 (65.5%)
Pauwels’ III	1 (5.8%)	16 (94.2%)^a^

^*^The odds ratio for optimal outcomes in the Pauwels’ II group was 8.57 (*p* = 0.025 using Fisher’s exact test)

^a^Note the high complication rate in the Pauwels’ III group

**Table 4:** Outcomes in the acceptable reduction group according to smoking, alcohol consumption, and diabetes

	Optimal outcomes	Complications	*P*-value^*^
Smoking	2 (18.1%)	10 (25.6%)	0.472
Alcohol consumption	0	11 (28.2%)	0.045
Diabetes mellitus	1 (9%)	7 (17.9%)	0.430

^*^P-values were calculated using Fisher’s exact test

## Discussion

**Fig. 1:**
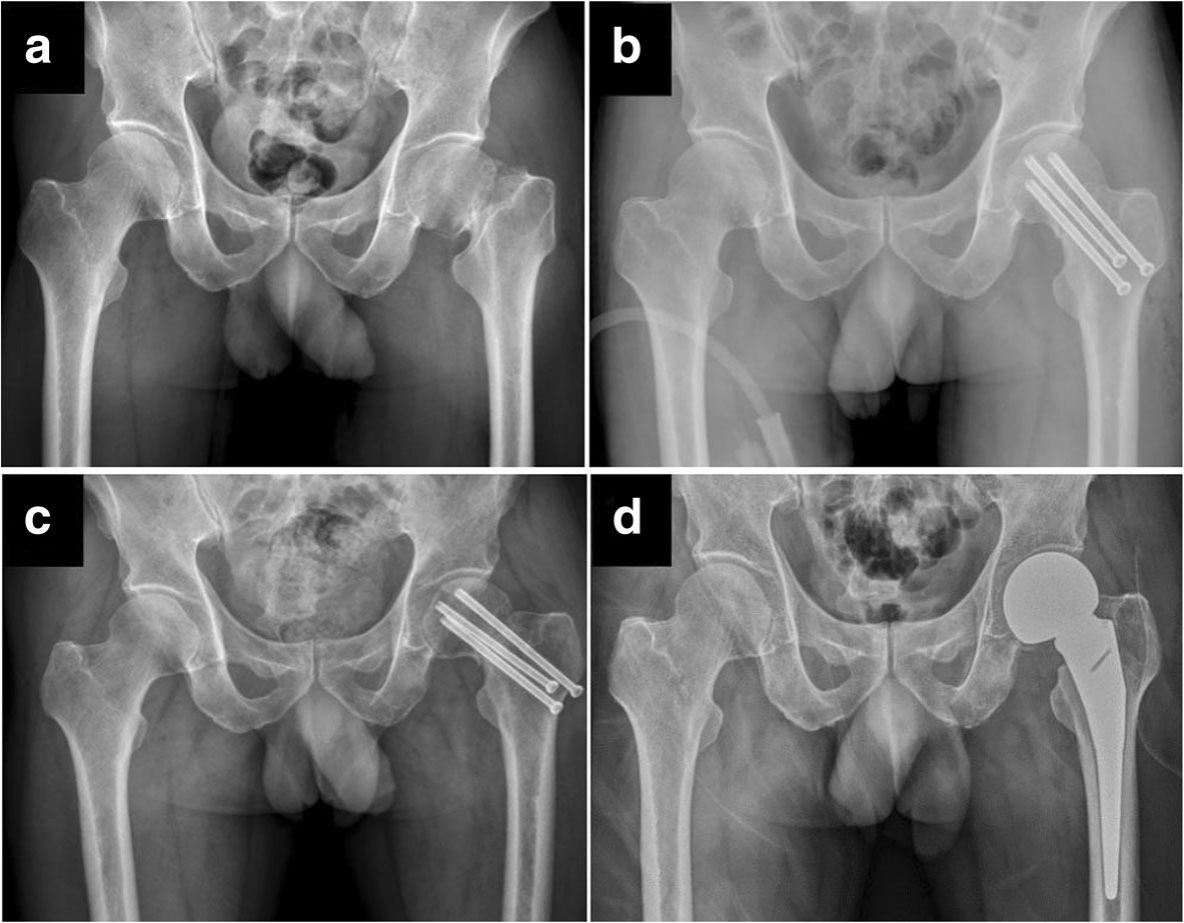
A typical clinical course of failed internal fixation in middle-aged patient. **a** A 56-year-old male patient with history of alcohol consumption sustained a left displaced femoral neck fracture. **b** Three cannulated screws were applied within 6 h and acceptable reduction was achieved. **c** Postoperative 6 months, there was still non-union with screw backout. **d** Screws were removed and hemiarthroplasty was performed. The case highlighted a common but catastrophic course of middle-aged patients with FNFs, even good reduction was achieved during primary fixation

Internal fixation is the gold standard treatment for non-displaced fractures in young and elderly patients, because it provides optimal outcomes [12, 13]. Our results support this approach, as 93.8% of that patient group experienced optimal outcome without complications. However, the failure rate for displaced fractures is much higher than that for non-displaced fractures, and displaced fractures are frequently complicated by AVN and non-union [3, 4]. Non-union of FNFs represents an early mechanical failure before bony union is achieved, and this complication is considered catastrophic because it necessitates revisional surgery (Fig. 1). Barnes et al. have reported an increased age-related risk of non-union [14], and Parker et al. have reported that their 50–60-year-old patients commonly experienced displacement (30%) and non-union (13.5%) [3]. In contrast, a study of young patients with displaced FNFs (15–50 years old) revealed a non-union rate of only 8%, and the authors concluded that this result was influenced by fracture displacement and reduction quality [15]. In comparison with the younger group, we observed a relatively high non-union rate (34%) among middle-aged patients in the present study, which might imply that a wide range of “young patients” is inadequate.

Although AVN is not an urgent problem, it can lead to chronic hip pain and subsequent arthroplasty. The incidence of AVN is higher among displaced FNFs than in undisplaced FNFs [3, 16]. Furthermore, a prospective study from 2009 revealed a relatively high rate of AVN among young patients (< 60 years old: 20.5%, > 80 years old: 12.5%) [4]. The benefit of decompression of the capsule to relieve pressure in femoral neck fractures remains unclear. This treatment is not completely supported in the current literature, and many studies reported no significant difference in patient outcomes with capsulotomy [17–20]. The present study revealed an AVN rate of 16% among patients with good reduction, although we failed to identify a significant association between AVN development and reduction quality.

Relatively minor complications, such as varus malunion and neck shortening, can also lead to unsatisfactory outcomes. Although it is generally not necessary to perform arthroplasty for varus malunion or shortening, problematic complications can emerge during long-term follow-up, such as hip impingement, loss of hip motion, arthrosis, and labral lesions. Concomitant screw back-out can also cause hip irritation and a limping gait. Zlowodzki et al. found, in an observational cohort study, that femoral neck shortening had the greatest adverse effect on the SF-36 physical functioning score [7], and Zielinski et al. reported that femoral neck shortening can impair gait velocity [21]. Thus, novel devices have been developed to prevent neck shortening, and implants with a locked side plate may be a solution to the fractured neck sliding along multiple cancellous screws. However, the use of locking devices for FNFs remains controversial, as a retrospective study from 2014 revealed catastrophic outcomes among patients who received locking devices, which may have been related to decreased micromotion at the fracture site [22]. In the present study, most patients with varus malunion or shortening developed an asymmetric gait and required walking aids or shoe lifts. Thus, even when fracture healing was achieved without non-union or AVN, patients still experienced complications because of femoral neck malunion or shortening.

Unlike sex, underlying disease, osteoporosis, and fracture pattern, adequate reduction and stable fixation are controllable factors in the treatment of displaced FNFs. Good reduction and adequate fixation are associated with successful treatment of young patients [23]. Haidukewych et al. reported that good reduction helped prevent AVN and non-union among 15–50-year-old patients [15]. Our study revealed that intraoperative acceptable reduction is considered a basic requirement for achieving optimal outcomes; none of our patients with unacceptable reduction achieved optimal outcomes. However, even acceptable reduction was achieved for displaced fractures, most of the cases (18 patients, 36%) subsequently underwent either bipolar hemiarthroplasty (13 cases) or total hip replacement (5 cases). In addition, 15 patients who experienced minor complications (e.g., varus malunion and neck shortening) experienced chronic hip pain and gait disturbance that necessitated the long-term use of walking aids or shoe lifts.

Pauwels’ type III fractures have a high complication rate because of the dominant vertical shearing force. Some clinicians suggest using a sliding hip screw for unstable fracture patterns. Deneka et al. reported that a sliding hip screw with a de-rotation screw provides superior outcomes for unstable fractures, compared to parallel cancellous screws [24]. In contrast, Clark et al. did not detect any difference between cases of subcapital fractures that were treated using a sliding hip screw or three cancellous screws in a triangular configuration [25]. A more recent study revealed inconclusive results regarding the “gold standard” of internal fixation for displaced FNFs, and the authors noted that fixation strength did not appear to be affected by subcapital or transervical patterns for the parallel cancellous screws or the sliding hip screw [26]. In the present study, all patients received parallel cancellous screws, and none received sliding hip screws. Nevertheless, we observed an extremely high complication rate for Pauwels’ type III fractures (16/17, 94.2%), even when acceptable reduction was achieved. The choice of the implant device depends on multiple factors and is not always clear; however, in hindsight, sliding hip screws and an additional de-rotation may provide more biomechanical stability to overcome vertical shearing force.

The present study revealed a notable association between optimal outcomes and Pauwels’ type II fractures (Table 3), as 91% (10/11) of the patients with optimal outcomes had Pauwels’ type II fractures. Furthermore, the odds ratio for optimal outcomes with Pauwels’ type II fractures was 8.67 (*p* = 0.025). This is likely because it is ideal to have the implant’s axis perpendicular to the fracture line, and we believe that the compression force from parallel screws in Pauwels’ type II fractures provides optimum efficiency (vs. type I and type III cases), which can promote fracture healing and bone consolidation. Furthermore, excessive alcohol consumption has been identified as a risk factor for suboptimal outcomes in previous reports [6], and our findings support this relationship among middle-aged patients. For example, 28% of the patients with complications in the acceptable reduction group reported excessive alcohol consumption, and none of the patients who achieved optimal outcomes reported excessive alcohol consumption. We believed that the concurrent physiological and psychological problems associated with alcohol abuse may lead to poor compliance and a high failure rate. Therefore, it is essential to consider the high risk in these patients, and other treatment options should be considered if surgery is indicated in these patients.

Besides internal fixation, primary arthroplasty has been advocated for displaced FNFs by many studies. Swart et al. found that primary total hip arthroplasty (THA) can be a cost-effective treatment for displaced FNFs among 45–65-year-old patients [27]. Rogmark et al. found that patient treated with arthroplasty have superior outcomes compared with those treated by internal fixation if there is no major complication [28]. Similarly, a national survey published in 2013 reported that patients treated with THA have less pain and better satisfaction compared with those treated with hemiarthroplasty or internal fixation [29]. However, the clinical judgement between internal fixation and arthroplasty is limited in countries with strict national health insurance programs. For example, Taiwanese patients are not covered for arthroplasty until the age of 60 years. Therefore, some middle-aged patients with high failure rate, such as Pauwels’ III fracture with excessive alcohol consumption, have no alternative but to receive a primary internal fixation and an unavoidable revision surgery.

The present study has several limitations. First, we did not include patients who received sliding hip screws, and it is possible that the specific implants could have influenced our findings, although there is no consensus regarding the ideal implant(s) for each fracture pattern. Furthermore, patients were not treated using open reduction, which may have increased the rate of unacceptable reductions and number of cases with suboptimal outcomes. Moreover, we identified several potentially significant prognostic factors, although the small sample size limited the power of the analyses and findings. Therefore, larger studies are needed to validate and expand on our findings.

In conclusion, the present study revealed an unacceptably high complication rate among middle-aged patients who underwent internal fixation for displaced FNFs, even when acceptable reduction was achieved. Thus, the overall rate of subsequent arthroplasty was high, especially in cases with non-union and AVN. Moreover, even when fracture union is achieved without AVN, varus malunion and shortening can lead to adverse outcomes. Although primary arthroplasty is another choice of treatment, the longevity of the prosthesis is still a major concern. If internal fixation is to be used for a displaced FNF, patient selection is essential to achieving optimal outcomes. Care must be taken to avoid selecting patients with excessive alcohol consumption, while successful internal fixation may be more likely for patients with Pauwels’ type II fracture.
